# Clostridium caseinilyticum sp. nov., a close relative of Clostridium tepidum and Clostridium sporogenes, isolated from spoiled cheese and silage

**DOI:** 10.1099/ijsem.0.006875

**Published:** 2025-08-07

**Authors:** Noam Shani, Miriam Zago, Hélène Berthoud, Daniel Marzohl, Emilie Michellod, Katia Gindro, Giorgio Giraffa, Emmanuelle Arias-Roth

**Affiliations:** 1 Competence Division Methods Development and Analytics, Research Group Fermentation Organisms, Agroscope, Bern, Switzerland; 2 Council for Agricultural Research and Economics, Research Centre for Animal Production and Aquaculture (CREA-ZA), Lodi, Italy; 3 Research Division Plant Protection, Research Group Mycology, Agroscope, Changins, Switzerland; 4 Research Division Food Microbial Systems, Research Group Cheese Quality, Cultures and Terroir, Agroscope, Bern, Switzerland

**Keywords:** *Clostridium caseinilyticum*, *Clostridium sporogenes*, *Clostridium tepidum*, food spoilage, cheese, anaerobic

## Abstract

Several *Clostridium* species are responsible for significant losses in the food industry. Here, a novel obligately anaerobic, spore-forming, motile bacterium, designated *Clostridium caseinilyticum* sp. nov., was isolated from hard and extra-hard cheeses with a defect consisting of a degradation of the cheese matrix. The type strain, FAM 1755^T^, was obtained from Swiss Emmentaler cheese in 1983, and additional strains CREA 4990 and FAM 27665 were isolated from Italian Grana Padano in 2022 and from Swiss silage in 2012, respectively. The rod-shaped, Gram-positive cells of FAM 1755^T^ measured 3–4.5 µm in length and 0.7–0.9 µm in width. Phylogenetic analyses of whole-genome and 16S rRNA gene sequences placed the new taxon close to *Clostridium tepidum* DSM 104389^T^ and to members of the *Clostridium botulinum* Group I, such as *Clostridium sporogenes* DSM 795^T^. Average nucleotide identity (ANI) values with related species were below the 95% prokaryotic species threshold, with the highest similarity to *C. tepidum* DSM 104389^T^ (91.4% ANI). The new species grew at a slightly lower temperature range (20–40 °C) than *C. tepidum* and *C. sporogenes* and was more halotolerant [0–5% (w/v) NaCl] than *C. tepidum*. Although genetically closer to *C. tepidum*, its growth preferences were more similar to those of *C. sporogenes*. It was catalase-, oxidase- and urease-negative, fermented glucose, and hydrolyzed gelatin but not aesculin. The polar lipid profile of FAM 1755^T^ included phosphatidylglycerol, diphosphatidylglycerol and phosphatidylethanolamine, accompanied by an unidentified aminophospholipid, several unidentified aminolipids, phospholipids and lipids. Major cellular fatty acids were C_18 : 1_ CIS 9, C_16 : 0_, C_14 : 0_, and C_18 : 1_ DMA. The genomic DNA G+C content was 27.4 mol%. Unlike *C. tepidum*, the new species consistently hydrolyzed milk proteins, a feature implicated in cheese spoilage. Consequently, we propose the name *C. caseinilyticum* sp. nov., with the type strain FAM 1755^T^ (=DSM 117478^T^=LMG 33232^T^=CCOS 2102^T^).

## Introduction

The genus *Clostridium* was first proposed by Prazmowski [1] and encompasses today 169 species with validly published and correct names, with *Clostridium butyricum* designated as the type species [2]. The genus has been divided into 19 phylogenetically distinct clusters, covering altogether a wide genetic diversity [3]. However, only species within cluster I are considered to be part of the genus *Clostridium sensu stricto* [4].

Most species belonging to the genus *Clostridium sensu stricto* are Gram-positive rods, are strictly anaerobic and form endospores [4]. This taxonomic group includes well-known pathogens, such as *Clostridium botulinum* and *Clostridium tetani*. Additionally, it comprises the most prevalent cheese defect-associated *Clostridium* species, including *Clostridium tyrobutyricum*, *C. butyricum*, *Clostridium beijerinckii* and *Clostridium sporogenes* [5]. Unlike the others, which produce gas through the butyric fermentation of lactate, *C. sporogenes* has been associated with defects of the cheese structure resulting from proteolysis. Furthermore, the co-occurrence of this species with *C. tyrobutyricum* has been shown to further exacerbate the cheese defects caused by the latter [6,7].

Recently, Carminati *et al*. described strains of the presumed *C. sporogenes* involved in an atypical defect of long-ripened Grana Padano PDO cheese [8]. In this cheese, the defect became apparent after 6–7 months of ageing and was characterized by the appearance of whitish spots within the interior sections. As the issue advanced, it frequently resulted in the development of irregular, sometimes relatively large voids, accompanied by an unpleasant odour. Interestingly, genotypic and phenotypic characterization of cheese isolates had shown the presence of two different prevalent strains of presumed *C. sporogenes*, suggesting that two separate genetic lines had been isolated from spoiled cheeses [8]. In Swiss Emmentaler PDO cheese, a similar defect, referred to as ‘putrificus’, has already been known in the first half of the twentieth century [9], and strains of presumed *C. sporogenes* have been isolated from affected cheese wheels.

Recent genome-based phylogenetic analyses revealed that the isolates involved in the atypical cheese defect belonged to a not-yet-described taxon related to, but distinct from, *Clostridium tepidum*, *Clostridium combesii*, *C. botulinum* and *C. sporogenes*. Subsequent comparisons with older strains preserved in the Agroscope Culture Collection revealed that some isolates previously classified as *C. sporogenes* should be reassigned to this new taxon. One strain, FAM 1755, isolated in 1983 from a Swiss Emmentaler cheese under the laboratory number 2d, was found to be highly similar to strains isolated recently, both from Grana Padano PDO and Emmentaler PDO.

Given the significant impact of *Clostridium* spp. on cheese quality, particularly the defects caused by *C. sporogenes*, it is crucial to accurately identify and classify these bacteria. Here, we describe *Clostridium caseinilyticum* sp. nov., a novel taxon isolated from spoiled cheese and from silage, through an exhaustive polyphasic approach involving genomic, biochemical and phenotypic analyses. The description of the new taxon is based on analyses of FAM 1755^T^, designated as the type strain, along with two more recent strains, CREA 4990 (isolated from Grana Padano PDO, Italy, 2022) [8] and FAM 27665 (isolated from silage, Switzerland, 2012). Furthermore, metabolic characteristics of the new taxon were evaluated to clarify its role in cheese spoilage.

Our identification of *C. caseinilyticum* sp. nov. as a contributor to cheese spoilage highlights the need for targeted monitoring and preventive strategies. Preventing this species from entering the processing line vehiculated by milk (e.g. screening raw milk and silage for the presence of proteolytic clostridia), along with adherence to good hygiene and production practices, could be the safest option to minimize the onset of the defect during cheese ripening. Furthermore, future research focusing on identifying the specific environmental factors that favour *C. caseinilyticum* sp. nov. growth would support the development of more effective preventive measures.

## Isolation, maintenance and testing of growth conditions

All strains discussed in this article and related information are listed in Table 1. The strains were obtained as part of various research projects investigating the microbiological causes of the ‘putrificus’ defect, often detected only after months of ripening, and were originally identified as *C. sporogenes*. FAM 27665 was isolated on differential reinforced clostridial medium agar (Merck) and purified by subculturing a single colony type on the same medium. CREA 4990 was isolated on reinforced clostridial agar (Oxoid) after 7 days of incubation in enriched milk and purified as described in [8]. Details about the isolation conditions of FAM 1755^T^ are not available. To ensure quality, it was purified following the same procedure as described for FAM 27665. Routine cultivation of the isolates was carried out at 30 °C in reinforced clostridial broth (RCM, Oxoid) under anaerobic conditions. The isolates were stored at −80 °C in RCM supplemented with 30% sterile skim milk as cryoprotectant (FAM 1755^T^ and FAM 27665) and in RCM with 15% glycerol (CREA 4990).

**Table 1.** Bacterial strains used in this study and their taxonomic affiliation, origin, year of isolation and GenBank accession numbers

Public culture collection strain no.*	Species†	Culture collection‡	Origin	Year of isolation	Original depositor; strain designation	GenBank assembly accession no.
DSM 117478^T^=LMG 33232^T^=CCOS 2102^T^	*C. caseinilyticum* sp. nov.	ACC	Emmental cheese (Switzerland)	1983	Grand, M.; 2d, **FAM 1755**	GCA_046127645.1
na	*C. caseinilyticum* sp. nov.	ACC	Silage (Switzerland)	2012	Aslan, B.; IS98, **FAM 27665**	GCA_046127635.1
na	*C. caseinilyticum* sp. nov.	CREA-ZA	Grana Padano (Italy)	2022	Carminati, D.; **CREA 4990**	GCA_031629375.1
**DSM 104389^T^**	*C. tepidum*	DSMZ	Bloated bottle of non-dairy protein shake (USA)	2016	Ma, G.; IEH 97212	GCA_002008345.1
**DSM 795^T^**	*C. sporogenes*	DSMZ	Soil (country of origin unknown)	1976	Peterson, E. C.; 388	GCA_000685115.1
**DSM 46279**	*C. caseinilyticum* sp. nov.	DSMZ	Unknown	Before 27 February 1991	Schloßberger; 15Rga	GCA_046127655.1

*Strain numbers in the public collections from which the strains were obtained or where they were deposited; the type strain of *C. caseinilyticum* sp. nov. (first row) is shown with the three public collections where it has been deposited.

‡Culture collections from which the strains were obtained for this study. ACC: Agroscope Culture Collection, Agroscope, Switzerland; CREA-ZA: Council for Agricultural Research and Economics, Research Centre for Animal Production and Aquaculture, Lodi, Italy; DSMZ: German Collection of Microorganisms and Cell Cultures GmbH, Braunschweig, Germany. The strain identifiers used in this study are indicated in bold.

FAM 1755^T^ has been deposited at the Culture Collection of Switzerland (CCOS), at the Belgian Culture Collection of Microorganisms and at the German Collection of Microorganisms and Cell Cultures (DSMZ), respectively, under the accession numbers CCOS 2102, LMG 33232 and DSM 117478.

Experiments to assess the range of temperature, pH and salt concentration required for growth were carried out using the modified medium 104b (PY+X) [10], with spore suspensions used as inoculum, as described previously [11]. Strains were grown in duplicates in microplates under anaerobic conditions, and mean values were reported. Growth was evaluated after 48 and 72 h by OD_600_ measurement using a BioTek Synergy H1 Multimode Microplate Reader (Agilent Technologies, CA, USA). Growth temperature was evaluated across a range of 10 °C–50 °C, with 5 °C intervals. The pH range for growth was evaluated between 4.0 and 9.5 with 0.5 pH unit intervals, and salt tolerance by growing the strains at NaCl concentrations across a range of 0–10% (w/v) with 1% intervals, as described previously [10].

## Phylogenetic analyses

Whole-genome sequences (WGSs) of FAM 1755^T^, FAM 27665 and DSM 46279 were generated using the Pacific Bioscience (PacBio) long-read technology on the Next Generation Sequencing Platform, University of Bern, Switzerland. The WGS of CREA 4990 was obtained using the Illumina short-read technology at Personal Genomics laboratories (Verona, Italy). The draft genomes were deposited in GenBank under accession numbers GCA_046127645.1 (FAM 1755^T^), GCA_031629375.1 (CREA 4990), GCA_046127635.1 (FAM 27665) and GCA_046127655.1 (DSM 46279). To control the purity of the genomes, the 16S rRNA gene of the strains was sequenced using the Sanger technology at Fasteris, Life Science Genesupport SA (Switzerland). The Sanger sequences of the 16S rRNA genes of FAM 1755^T^, CREA 4990, FAM 27665 and DSM 46279 were deposited in GenBank under accession numbers PV031525, PV031526, PV031528 and PV031527, respectively.

**Fig. 1.**
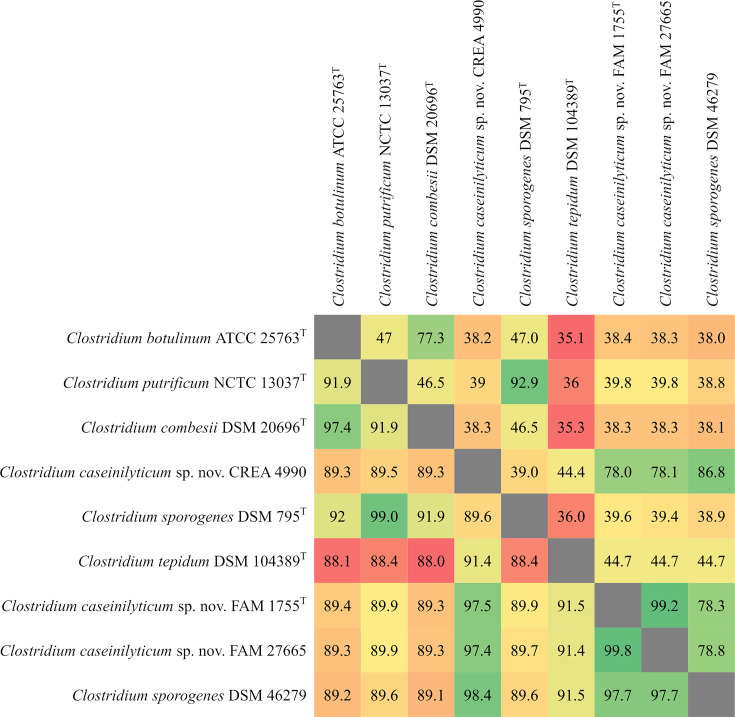
Genome-to-genome comparisons of *C. caseinilyticum* sp. nov. FAM 1755^T^, CREA 4990, FAM 27665, DSM 46279 and their closest relative species. The ANI values are displayed in the lower triangular part and the dDDH values (d_4_ value of TYGS) in the upper one. *C. sporogenes* DSM 46279 was added to the analysis after its MSP indicated that it should be re-classified as *C. caseinilyticum* sp. nov. and is discussed separately in the main text.

Phylogenetic analyses were performed using the Type (Strain) Genome Server (TYGS), a free bioinformatics platform available under https://tygs.dsmz.de, for a whole genome-based taxonomic analysis [12]. The analysis also made use of recently introduced methodological updates and features [13]. Information on nomenclature, synonymy and associated taxonomic literature was provided by TYGS’s sister database, the List of Prokaryotic names with Standing in Nomenclature (available at https://lpsn.dsmz.de) [13]. The results provided by the TYGS indicated that FAM 1755^T^, FAM 27665 and CREA 4990 probably belong to a new species. Digital DNA–DNA hybridization (dDDH) values between the *C. caseinilyticum* sp. nov. strains ranged between 78.0 and 99.2% (d_4_ value of TYGS), whereas they ranged between 44.4 and 44.7% with *C. tepidum* DSM 104389^T^ (Fig. 1). dDDH with other species was below 40%. Additionally, the average nucleotide identity (ANI) values calculated using fastANI [14] ranged between 97.4 and 99.8% between the three *C. caseinilyticum* sp. nov. strains, and below 93% when compared with all other strains, *C. tepidum* DSM 104389^T^ being the closest relative (91.4% ANI). Thus, compared with validly described species, both dDDH and ANI values (Fig. 1) were below the species delineation values of 70 and 95 %, respectively [14,15], confirming that *C. caseinilyticum* sp. nov. is a distinct species.

To place *C. caseinilyticum* sp. nov. in a phylogenetic tree, the 16S rRNA gene sequences were extracted from the genomes of FAM 1755^T^, CREA 4990, FAM 27665 and DSM 46279 and deposited in GenBank under accession numbers PV016861, PV016862, PV016863 and PV016864, respectively. Alignments of 16S rRNA gene sequences were generated using MAFFT v. 7.520 with the default parameters [16]. The maximum-likelihood tree was calculated on the online IQ-TREE (multicore version 2.3.6) web server with parameter setting ‘-m TEST -bb 1000 -alrt 1000’ [17,18] and visualized with the Interactive Tree Of Life (iTOL) v.7 [19]. Additionally, WGS were processed using OrthoFinder v. 2.5.5 [20] to generate a second maximum-likelihood phylogenetic tree, visualized with iTOL.

**Fig. 2.**
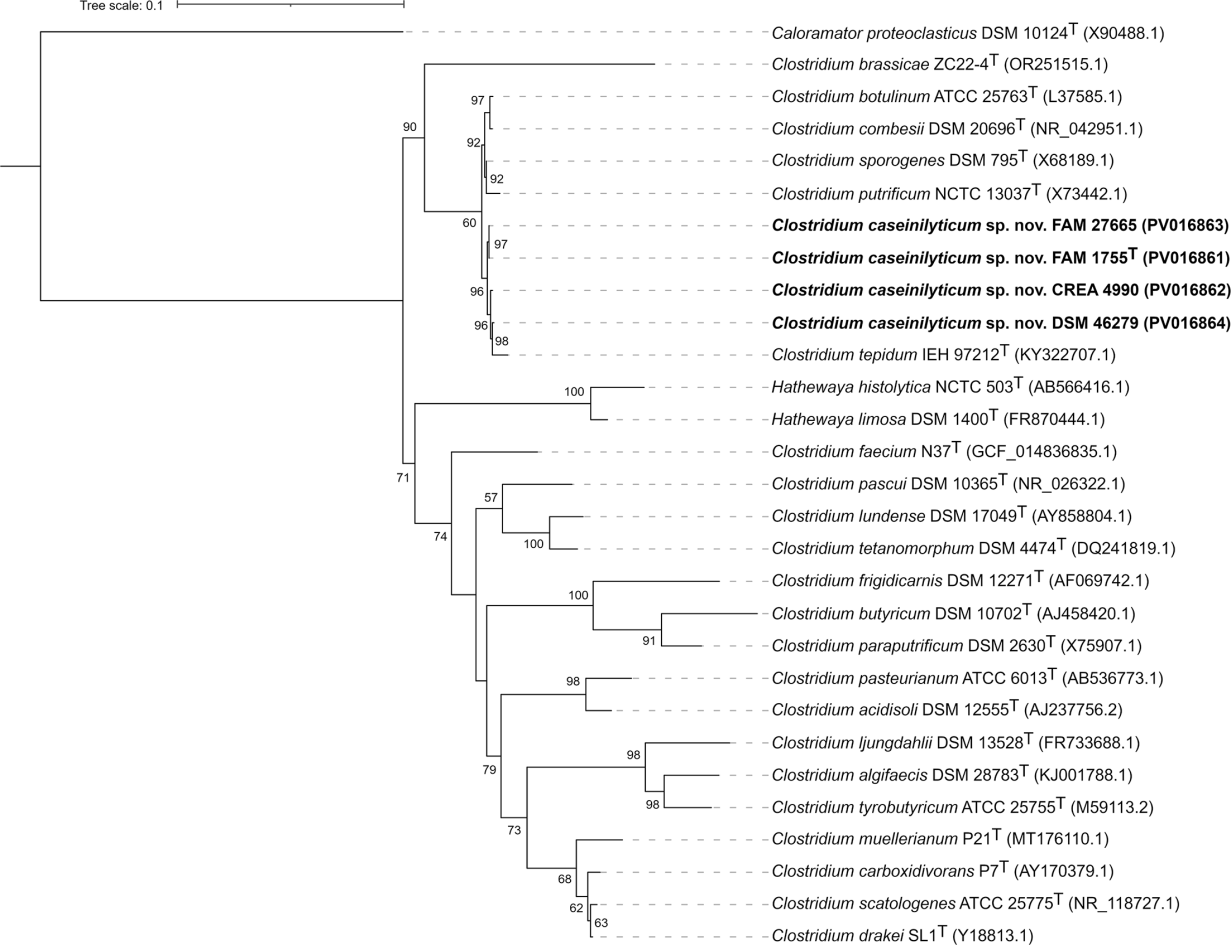
Phylogenetic tree of 16S rRNA gene sequences, displaying the position of *C. caseinilyticum* sp. nov. strains FAM 1755^T^, CREA 4990 and FAM 27665, together with other members of the genus *Clostridium* and with some other members of the family *Clostridiaceae*. DSM 46279, referenced as *C. sporogenes* in public databases, was also placed in the tree, showing its proximity to *C. caseinilyticum* sp. nov. GenBank accession numbers are shown in brackets. Bootstrap values>50 are given as node labels. Bar: 0.1 substitutions per site.

**Fig. 3.**
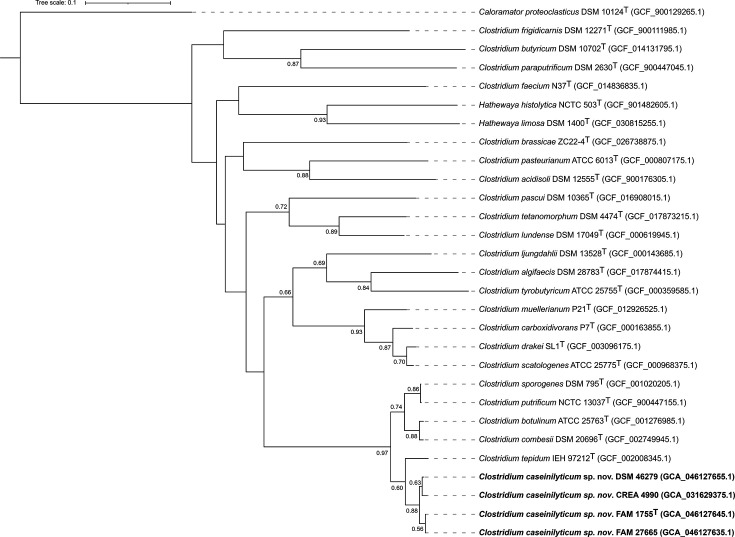
Phylogenetic tree of WGSs, displaying the position of *C. caseinilyticum* sp. nov. strains FAM 1755^T^, CREA 4990 and FAM 27665, together with other members of the genus *Clostridium* and with some other members of the family *Clostridiaceae*. DSM 46279, referenced as *C. sporogenes* in public databases, was also placed in the tree, showing its proximity to *C. caseinilyticum* sp. nov. GenBank accession numbers are shown in brackets. Bootstrap values>0.5 are given as node labels. Bar: 0.1 substitutions per site.

Based on the analysis of the 16S rRNA gene, the strains of the new taxon were placed within a tight cluster encompassing *C. tepidum*, *C. sporogenes*, *Clostridium putrificum*, *C. combesii* and *C. botulinum* (Fig. 2). Based on WGS analysis, however, the *C. caseinilyticum* sp. nov. strains formed a distinct cluster, with *C. tepidum* DSM 104389^T^ as the closest relative (Fig. 3).

## MALDI-TOF spectra

Mass spectral profiles (MSPs) of FAM 1755^T^, CREA 4990 and FAM 27665, and of the type strains of the closest species that were not yet available in the BDAL v11.0 library, were generated following Bruker’s recommendations. The protein extracts were analysed on a MicroFlex^™^ LT/SH mass spectrometer (Bruker Daltonics, Bremen, Germany). Mass spectra were acquired with the FlexControl v. 3.4 (Build 206.94). Each spectrum was summed up to 240 laser shots (40 laser shots/position×6 different positions). Each MSP was generated using at least 20 single spectra and added to our local database. To check the validity of the MSPs, FAM 1755^T^ was subsequently submitted to routine MALDI-TOF analysis as described previously [21]. The spectrum was analysed with the MBT Compass software v.1.4 (Bruker Daltonics, Inc., Billerica, MA, USA) and by Realtime Classification Biotyper MBT RUO 3.1 with the BDAL v11.0 library complemented with the new MSP. Unsurprisingly, the best match was the created reference MSP FAM 1755^T^. All *Clostridium* spp. available in the BDAL v11.0 library provided scores below 1.70, except for a *C. sporogenes* strain, DSM 46279, which displayed a high score (2.27). We thus sequenced the genome of the latter (GenBank accession no. GCA_046127655.1) and compared it to the genomes of different *Clostridium* spp. The dDDH values between DSM 46279 and *C. caseinilyticum* sp. nov. strains ranged from 76.3 to 86.8, with ANI values between 97.7 and 98.4. In contrast, its comparison to the closest related species, *C. tepidum*, yielded dDDH and ANI values of 44.7 and 91.5, respectively. These results confirmed that DSM 46279 should be reclassified as *C. caseinilyticum* sp. nov.

## Phenotypic features

**Fig. 4.**
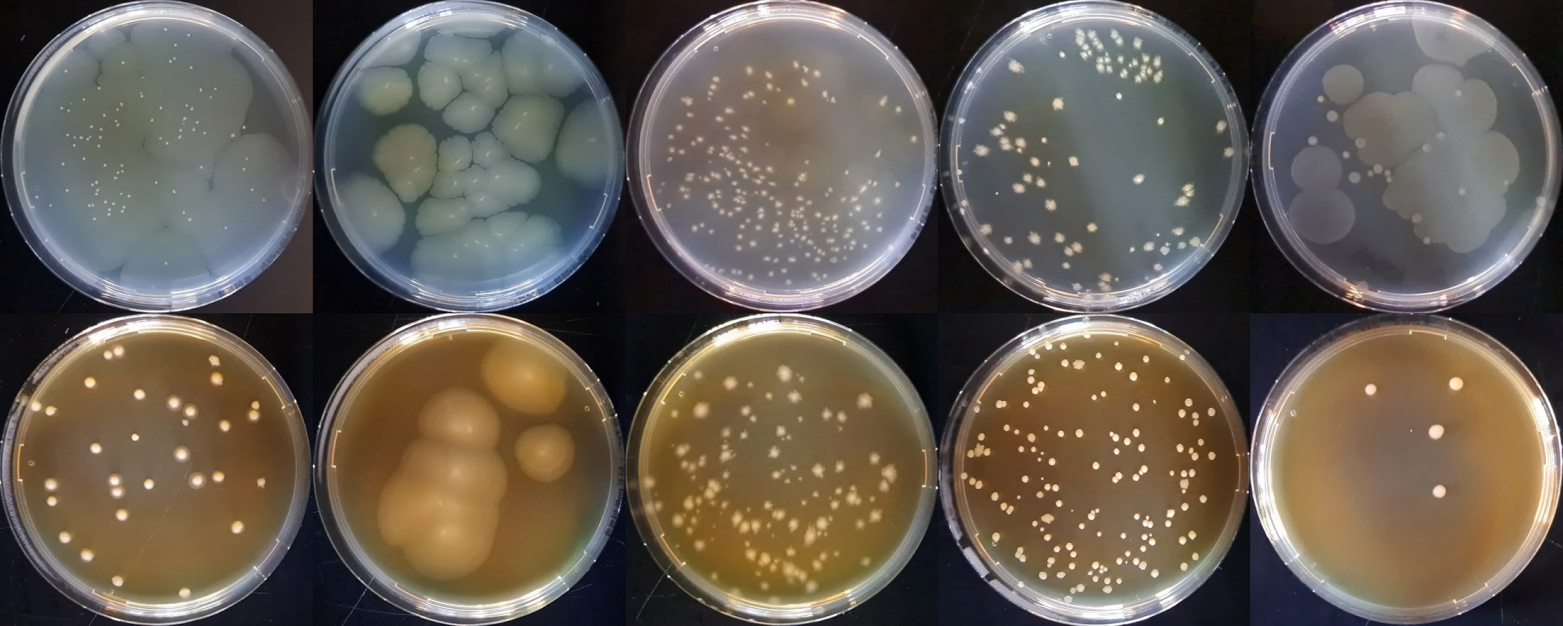
Colony morphology of (from left to right) *C. caseinilyticum* sp. nov. FAM 1755^T^, CREA 4990 and FAM 27665 on RCM (upper row) and MRS (lower row) agar plates, and comparison with *C. sporogenes* DSM 795^T^ and *C. tepidum* DSM 104389^T^.

Colony morphologies of *C. caseinilyticum* sp. nov. FAM 1755^T^, CREA 4990 and FAM 27665 were assessed on RCM and MRS agar plates after 96 h incubation at 30 °C in anaerobic conditions and compared to *C. tepidum* DSM 104389^T^ and *C. sporogenes* DSM 795^T^ grown in the same conditions. *C. caseinilyticum* sp. nov. FAM 1755^T^, CREA 4990 and FAM 27665 formed small irregular colonies on RCM, widely spreading on the agar. The colonies of FAM 27665 on this medium had a slight rhizoid edge (Fig. 4). On MRS, only CREA 4990 formed widely spreading colonies. On this medium, colonies of FAM 1755^T^ were in the majority round-shaped with a regular margin, some of them slightly spreading. The colonies of FAM 27665, on the other hand, were irregular in shape with a slight rhizoid edge.

FAM 1755^T^ and DSM 104389^T^ exhibited two distinct colony morphologies on RCM (round-shaped or widely spreading). Multiple colonies of each type were identified using MALDI-TOF to rule out contamination.

None of the three *C. caseinilyticum* sp. nov. strains exhibited haemolysis on blood agar (data not shown).

**Fig. 5.**
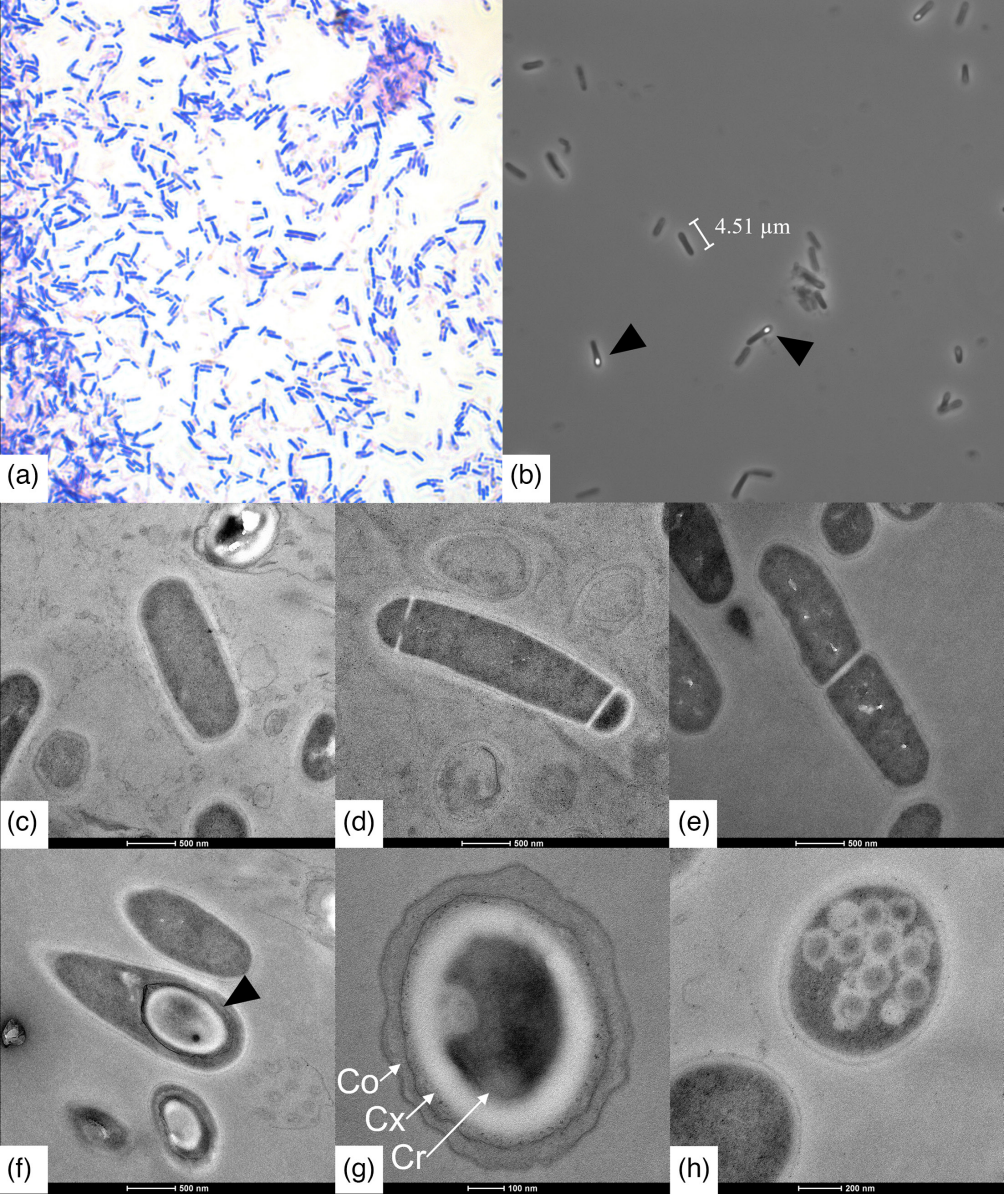
Microscopic images of *C. caseinilyticum* sp. FAM 1755^T^. Gram-stained cells (a); phase-contrast microscopic image of rod-shaped cells with endospores visible in some of them (black arrowheads; (b); transmission electron microscopic images of a single cell (**c**); dividing cells (**d, e**); a terminal endospore (black arrowhead, (**f**); a free spore (**g**) with a view of the core (Cr), the cortex (Cx) and the coat (Co); and undefined cell inclusions (**h**).

All three *C. caseinilyticum* sp. nov. strains had motile cells, as evaluated by the stab inoculation method [22] in RCM containing 0.3% agar. The cells of FAM 1755^T^ were Gram-positive rods, about 3–4.5 µm long and 0.7–0.9 µm wide (Fig. 5). All three strains were sporulating, with oval terminal spores forming little dwelling of the cells. Some cells displayed unidentified inclusions visible under the transmission electron microscope.

## Growth preferences

Results of the growth preferences are summarized in Table 2

**Table 2.** Phenotypic differences among *C. caseinilyticum* sp. nov. FAM 1755^T^ (1), CREA 4990 (2) and FAM 27665 (3), compared with *C. sporogenes* DSM 795^T^ (4) and *C. tepidum* DSM 104389^T^ (5)

Characteristic*	1	2	3	4	5
Colony morphology on RCM	Small; irregularly circular; widely spreading	Small; irregular; slightly elongated; widely spreading	Small, irregular; slight rhizoid edge; widely spreading	Medium-sized; irregular; slightly rhizoid edge; **not spreading**	Medium-sized; **regularly round-shaped**; widely spreading
Temperature range (°C)	20–40	25–40	25–40	25–45	30–45
(optimum)	(25–30)	(25–30)	(25–30)	(25–30)	(**30–35**)
pH range	5.5–9.5	5.5–9.5	5.5–9.5	5.5–9.5	**6.0–9.5**
(optimum)	(5.5–6.0)	(5.5–6.0)	(6.0)	(6.0–8.5)	(**9.0**–**9.5**)
Salt tolerance (%)	0–5	0–5	**0–4**	0–5	**0–2**
(optimum)	(1–2)	(1–2)	(1–2)	(**4**)	(**0**)
Fermentation of cellobiose	+	+	−	−	−
Fermentation of maltose	−	−	+	+	+
Fermentation of mannose	−	+	+	−	−
Fermentation of melezitose	−	+	−	−	−
Fermentation of trehalose	+	+	−	+	+
Casein hydrolysis	+	+	+	+	−

*Phenotypic characteristics are standard criteria that complement genomic analyses in differentiating closely related species. The features showing the greatest divergence between species are indicated in bold: colony morphology, growth temperature optimum, pH range and salt tolerance.

Growth was observed between 20 °C and 40 °C (optimum 25–30 °C) for FAM 1755^T^ and CREA 4990, and between 25 °C and 40 °C (optimum 25–30 °C) for FAM 27665. In our experiments, the growth temperature ranges for *C. sporogenes* DSM 795^T^ and *C. tepidum* DSM 104389^T^ were 25–45 °C (optimum 25–30 °C) and 30–45 °C (optimum 30–35 °C), respectively.

*C. caseinilyticum* sp. nov. FAM 1755^T^, CREA 4990 and FAM 27665 grew within a pH range of 5.5–9.5. The optimal pH was 5.5–6.0 for FAM 1755 and CREA 4990, and 6.0 for FAM 27665. *C. sporogenes* DSM 795^T^ grew between pH 5.5 and 9.5, with an optimum at 6.0–8.5, while *C. tepidum* DSM 104389^T^ grew at pH 6.0–9.5, with an optimum at 9.0–9.5.

Regarding the salt tolerance, growth was observed between 0 and 5% NaCl (optimum 1–2 %) for FAM 1755^T^ and CREA 4990, and between 0 and 4% (optimum 1–2 %) for FAM 27665. *C. sporogenes* DSM 795^T^ grew at salt concentrations between 0 and 5% (optimum around 4%), while *C. tepidum* DSM 104389^T^ grew between 0 and 2% (optimum around 0%).

The growth preferences of *C. caseinilyticum* sp. nov. are more similar to those of *C. sporogenes* than *C. tepidum*, suggesting that the two former species thrive in comparable environments.

## Biochemical reactions

Classical biochemical reactions were analysed using API 20A test strips (bioMérieux) and Biolog AN microplate (Biolog Inc.) according to the manufacturer’s instructions. The results are presented in Table 2. All strains of *C. caseinilyticum* sp. nov. were able to ferment glucose and unable to (i) ferment lactose, raffinose, rhamnose, salicin, mannitol, sorbitol and sucrose and (ii) hydrolyze aesculin. Fermentation of cellobiose, maltose, mannose, melezitose and trehalose appeared to be strain-specific (Table 2). All strains were catalase-, oxidase- and urease-negative.

**Fig. 6.**
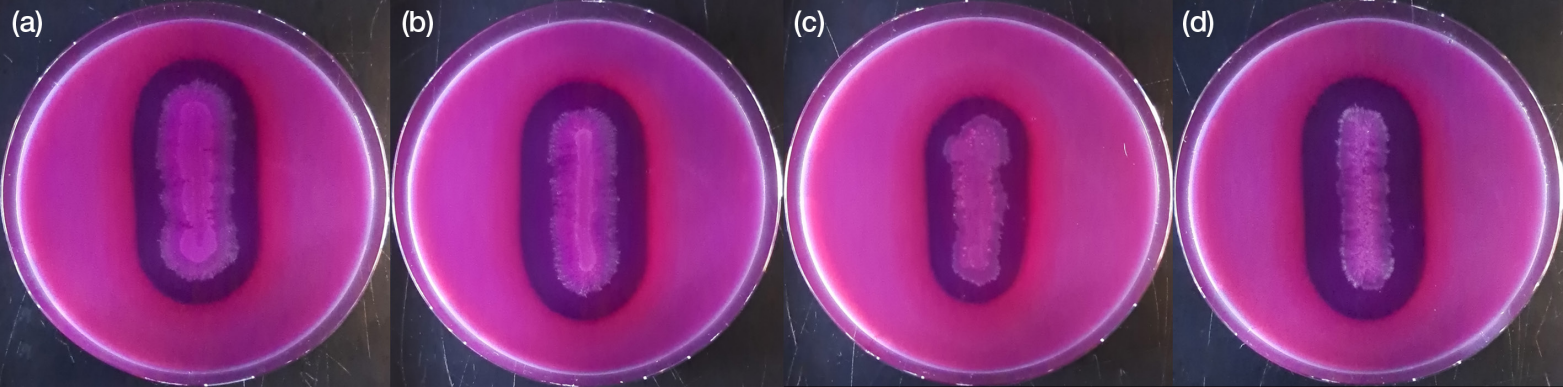
Casein degradation on skim milk agar supplemented with bromocresol purple. *C. caseinilyticum* sp. nov. FAM 1755^T^ (a), *C. caseinilyticum* sp. nov. CREA 4990 (b), *C. caseinilyticum* sp. nov. FAM 27665 (c) and *C. sporogenes* DSM 795^T^ (d). *C. tepidum* DSM 104389^T^ was unable to grow on skim milk agar.

The ability of the strains to hydrolyze casein was evaluated using the casein hydrolysis test modified from [23]. Briefly, strains were streaked on skim milk agar containing 28 g l^−1^ skim milk powder, 2.5 g l^−1^ yeast extract (Thermo Fisher Scientific), 5 g l^−1^ tryptone (Thermo Fisher Scientific), 1 g l^−1^ glucose (Merck), 0.5 g l^−1^ cysteine (Sigma-Aldrich) and 15 g l^−1^ agar (Oxoid). The plates contained either bromocresol purple or bromothymol blue as indicators of acidification and alkalization of the medium, respectively. The pH was adjusted to 7.0±0.2. The plates were incubated 48 h at 30 °C. Growth on skim milk agar was accompanied by a degradation of casein for both *C. sporogenes* DSM 795^T^ and *C. caseinilyticum* sp. nov. FAM 1755^T^, CREA 4990 and FAM 27665, with no indication of either an acidification or an alkalization of the medium (Fig. 6). *C. tepidum* DSM 104389^T^ was unable to grow on the skim milk agar (data not shown).

## Chemotaxonomy

Chemotaxonomy is mainly recommended for descriptions of new genera [24]. However, it can also provide additional bacterial taxonomy resolution to differentiate genetically closely related species.

Analyses of the peptidoglycan, cellular fatty acids and polar lipids were carried out by DSMZ Services, Leibniz-Institut DSMZ – Deutsche Sammlung von Mikroorganismen und Zellkulturen GmbH, Braunschweig, Germany.

The deduced peptidoglycan type was A1γ *meso*-Dpm-direct.

The polar lipid profile of FAM 1755^T^ consisted of phosphatidylglycerol, diphosphatidylglycerol and phosphatidylethanolamine, accompanied by an unidentified aminophospholipid, several unidentified aminolipids, phospholipids and lipids (Fig. S1, available in the online Supplementary Material).

For the analysis of the cellular fatty acids, *C. caseinilyticum* sp. nov. FAM 1755^T^, *C. sporogenes* DSM 795^T^ and *C. tepidum* DSM 104389^T^ were cultivated in modified PYG medium (DSMZ medium M104) at 37 °C for 24 h. Cellular fatty acids were first converted into fatty acid methyl esters (FAMEs) by saponification, methylation and extraction according to Sasser [25]. Mixtures of FAMEs were then separated by GC and detected by a flame ionization detector. Fatty acids were identified by GC-MS on an Agilent GC-MS 7000D system [26]. The cellular fatty acid (CFA) composition of FAM 1755^T^ was similar to that of *C. sporogenes* DSM 795^T^, but the CFA proportions differed (Table 3). The fatty acid composition of *C. tepidum* DSM 104389^T^ was different from both *C. caseinilyticum* sp. nov. FAM 1755^T^ and *C. sporogenes* DSM 795^T^, with some fatty acids only present (C_18 : 1_ TRANS 11) or absent (C_14 : 1_ DMA, C_15 : 0_) in the former one.

**Table 3.** Proportions of the major CFAs of *C. caseinilyticum* sp. nov. FAM 1755^T^ (1), *C. sporogenes* DSM 795^T^ (2) and *C. tepidum* DSM 104389^T^ (3). Only fatty acids with a proportion >1% in at least one of the four species are represented. nd: not detected

Fatty acids (%)	1	2	3
C_14 : 0_ ALDE	0.7	1.5	0.4
iso-C_14 : 0_	1.4	0.3	0.4
C_14 : 0_	13.9	14.5	4.4
C_14 : 1_ DMA	0.5	1.4	nd
C_14 : 0_ DMA	2.5	6.0	1.1
C_15 : 0_	2.1	2.6	nd
iso-C_16 : 0_	1.2	0.9	0.3
C_16 : 1_ CIS 9	1.9	1.6	1.0
C_16 : 0_	14.5	16.2	9.2
C_16 : 1_ DMA	2.3	4.8	0.6
C_16 : 0_ DMA	0.8	1.5	0.3
C_18 : 1_ CIS 9	33.4	21.9	51.6
C_18 : 1_ CIS 11	5.0	4.8	5.5
C_18 : 1_ TRANS 11	nd	nd	2.5
C_18 : 0_	1.4	2.4	1.8
C_18 : 1_ DMA	11.2	9.8	15.8
C_19 : 1_ cyclo 9,10	2.3	1.9	3.2

Although genetically close to *C. tepidum* DSM 104389^T^, *C. caseinilyticum* sp. nov. FAM 1755^T^ exhibited a CFA profile closer to *C. sporogenes* DSM 795^T^, confirming that the new taxon is distinct from *C. tepidum*.

## Description of *Clostridium caseinilyticum* sp. nov.

*Clostridium caseinilyticum* sp. nov. [ca.se.i.ni.ly'ti.cum. N.L. neut. n. *caseinum*, casein; N.L. masc. adj. *lyticus* (from Gr. masc. adj. *lytikos*), dissolving; N.L. neut. adj. *caseinilyticum*, casein-dissolving].

Cells are Gram-stain-positive, strictly anaerobic, motile, spore-forming, isolated or pairwise rods, ~3–4.5×0.7–0.9 µm in size. Endospores are oval and terminal, causing little swelling of the cells. Colonies are beige or whitish, regularly or irregularly edged, spreading or swarming on RCM and non-haemolytic on blood agar. Growth in RCM occurs from 20 to 40 °C, with 0–5% initial NaCl concentration at 30 °C, in a range of initial pH between 5.5 and 9.5. Optimal growth occurs at about 25–30 °C, with about 1–2% initial NaCl concentration. Cells are negative for catalase, oxidase and urease. They do not hydrolyze aesculin and do not produce indole. They ferment glucose and hydrolyze gelatin, and the fermentation of cellobiose, maltose, mannose, melezitose and trehalose is strain-specific. They do not ferment lactose, raffinose, rhamnose, salicin, mannitol, sorbitol and sucrose and do not hydrolyze aesculin. The predominant fatty acids are C_18 : 1_ CIS 9, C_16 : 0_, C_14 : 0_ and C_18:1_ DMA. The polar lipid profile consists of phosphatidylglycerol, diphosphatidylglycerol and phosphatidylethanolamine, accompanied by an unidentified aminophospholipid, several unidentified aminolipids, phospholipids and lipids. The peptidoglycan type is A1γ meso-Dpm-direct.

The type strain is FAM 1755^T^ (=CCOS 2102^T^=DSM 117478^T^=LMG 33232 ^T^), isolated in 1983 from a Swiss Emmentaler cheese presenting the so-called ‘putrificus’ defect. The genomic DNA G+C content of the type strain is 27.4 mol%, which is in the range usually observed in other *Clostridium* species [4], but higher than that of *C. tepidum* (26.7–26.9 mol%) and *C. sporogenes* (26.0 mol%), consistent with its taxonomic distinction.

The GenBank accession numbers of the draft genome sequences of strains FAM 1755^T^, CREA 4990, FAM 27665 and DSM 46279 are GCA_046127645.1, GCA_031629375.1, GCA_046127635.1 and GCA_046127655.1, respectively. The GenBank accession numbers of the 16S rRNA gene Sanger sequences of strains FAM 1755^T^, CREA 4990, FAM 27665 and DSM 46279, used for the control of genome purity, are PV031525, PV031526, PV031528 and PV031527, respectively. The accession numbers of the 16S rRNA gene sequences of strains FAM 1755^T^, CREA 4990, FAM 27665 and DSM 46279, extracted from genomic sequences and used for computing the 16S rRNA gene phylogenetic tree, are PV016861, PV016862, PV016863 and PV016864.

## Supplementary material

10.1099/ijsem.0.006875 Uncited Fig. S1.

## Abbreviations

ANI: average nucleotide identity
CCOS: Culture Collection of Switzerland
CFA: cellular fatty acid
dDDH: digital DNA–DNA hybridization
FAMEs: fatty acid methyl esters
iTOL: Interactive Tree Of Life
MSPs: mass spectral profiles
RCM: reinforced clostridial broth
TYGS: Type (Strain) Genome Server
WGSs: whole-genome sequences

## References

[1] Prazmowski A (1880). Untersuchungen Über Die Entwickelungsgeschichte Und Fermentwirkung Einiger Bacterien-Arten.

[2] Parte AC, Sardà Carbasse J, Meier-Kolthoff JP, Reimer LC, Göker M (2020). List of Prokaryotic names with Standing in Nomenclature (LPSN) moves to the DSMZ. Int J Syst Evol Microbiol.

[3] Collins MD, Lawson PA, Willems A, Cordoba JJ, Fernandez-Garayzabal J (1994). The phylogeny of the genus *Clostridium*: proposal of five new genera and eleven new species combinations. Int J Syst Bacteriol.

[4] Rainey FA, Hollen BJ, Small AM, Whitman WB, DeVos P, Dedysh S, Hedlund B, Kämpfer P (2015). Bergey’s Manual of Systematics of Archaea and Bacteria.

[5] Doyle CJ, Gleeson D, Jordan K, Beresford TP, Ross RP (2015). Anaerobic sporeformers and their significance with respect to milk and dairy products. Int J Food Microbiol.

[6] Goudkov AV, Sharpe ME (1965). Clostridia in dairying. J Appl Microbiol.

[7] Le Bourhis AG, Doré J, Carlier JP, Chamba JF, Popoff MR (2007). Contribution of *C. beijerinckii* and *C. sporogenes* in association with *C. tyrobutyricum* to the butyric fermentation in emmental type cheese. Int J Food Microbiol.

[8] Carminati D, Bonvini B, Rossetti L, Mariut M, Zago M (2024). Identification and characterization of the microbial agent responsible of an alteration in spoiled, Grana Padano cheese during ripening. Food Control.

[9] Kürsteiner J (1932). Vortrag mit lichtbildern und demonstrationen anlässlich des besuches der versuchsanstalt liebefeld durch den mittelländischen käserverein am 30.12.1931. Schweizerisches Zentralblatt für Milchwirtschaft.

[10] Dobritsa AP, Kutumbaka KK, Werner K, Wiedmann M, Asmus A (2017). *Clostridium tepidum* sp. nov., a close relative of *Clostridium sporogenes* and *Clostridium botulinum* group I. Int J Syst Evol Microbiol.

[11] Storari M, Kulli S, Wüthrich D, Bruggmann R, Berthoud H (2016). Genomic approach to studying nutritional requirements of *Clostridium tyrobutyricum* and other *Clostridia* causing late blowing defects. Food Microbiol.

[12] Meier-Kolthoff JP, Göker M (2019). TYGS is an automated high-throughput platform for state-of-the-art genome-based taxonomy. Nat Commun.

[13] Meier-Kolthoff JP, Carbasse JS, Peinado-Olarte RL, Göker M (2022). TYGS and LPSN: a database tandem for fast and reliable genome-based classification and nomenclature of prokaryotes. Nucleic Acids Res.

[14] Jain C, Rodriguez-R LM, Phillippy AM, Konstantinidis KT, Aluru S (2018). High throughput ANI analysis of 90K prokaryotic genomes reveals clear species boundaries. Nat Commun.

[15] Auch AF, von Jan M, Klenk H-P, Göker M (2010). Digital DNA-DNA hybridization for microbial species delineation by means of genome-to-genome sequence comparison. Stand Genomic Sci.

[16] Katoh K, Misawa K, Kuma K, Miyata T (2002). MAFFT: a novel method for rapid multiple sequence alignment based on fast fourier transform. Nucleic Acids Res.

[17] Nguyen L-T, Schmidt HA, von Haeseler A, Minh BQ (2015). IQ-TREE: a fast and effective stochastic algorithm for estimating maximum-likelihood phylogenies. Mol Biol Evol.

[18] Trifinopoulos J, Nguyen L-T, von Haeseler A, Minh BQ (2016). W-IQ-TREE: a fast online phylogenetic tool for maximum likelihood analysis. Nucleic Acids Res.

[19] Letunic I, Bork P (2024). Interactive Tree of Life (iTOL) v6: recent updates to the phylogenetic tree display and annotation tool. Nucleic Acids Res.

[20] Emms DM, Kelly S (2015). OrthoFinder: solving fundamental biases in whole genome comparisons dramatically improves orthogroup inference accuracy. Genome Biol.

[21] Shani N, Oberhaensli S, Berthoud H, Schmidt RS, Bachmann HP (2021). Antimicrobial susceptibility of *Lactobacillus delbrueckii* subsp. lactis from milk products and other habitats. Foods.

[22] Tittsler RP, Sandholzer LA (1936). The use of semi-solid agar for the detection of bacterial motility. J Bacteriol.

[23] Franciosi E, De Sabbata G, Gardini F, Cavazza A, Poznanski E (2011). Changes in psychrotrophic microbial populations during milk creaming to produce grana trentino cheese. Food Microbiol.

[24] Vandamme P, Sutcliffe I (2021). Out with the old and in with the new: time to rethink twentieth century chemotaxonomic practices in bacterial taxonomy. Int J Syst Evol Microbiol.

[25] Sasser M (1990). Identification of bacteria by gas chromatography of cellular fatty acids. MIDI Technical Note.

[26] Vieira S, Huber KJ, Neumann-Schaal M, Geppert A, Luckner M (2021). *Usitatibacter rugosus* gen. nov., sp. nov. and *Usitatibacter palustris* sp. nov., novel members of *Usitatibacteraceae fam*. nov. within the order *Nitrosomonadales* isolated from soil. Int J Syst Evol Microbiol.

