# Supplementary material for: Clostridium caseinilyticum sp. nov., a close relative of Clostridium tepidum and Clostridium sporogenes, isolated from spoiled cheese and silage
Source: Int J Syst Evol Microbiol. 2025 Aug 7;75(8):006875. doi: 10.1099/ijsem.0.006875 (PMC12451625; doi:10.1099/ijsem.0.006875)
Supplement: Uncited Fig. S1. [file ijsem-75-06875-s001.pdf]

Supplementary material for :

***Clostridium caseinilyticum* sp. nov., a close relative of *Clostridium tepidum* and *Clostridium sporogenes*, isolated from spoiled cheese and silage**

Noam Shani*, Miriam Zago, Hélène Berthoud, Daniel Marzohl, Emilie Michellod, Katia Gindro, Giorgio Giraffa and Emmanuelle Arias-Roth

International Journal of Systematic and Evolutionary Microbiology

*** Correspondence:**

Noam Shani
Research Group Fermenting Organisms
Agroscope
CH-3003 Bern, Switzerland
e-mail: noam.shani@agroscope.admin.ch

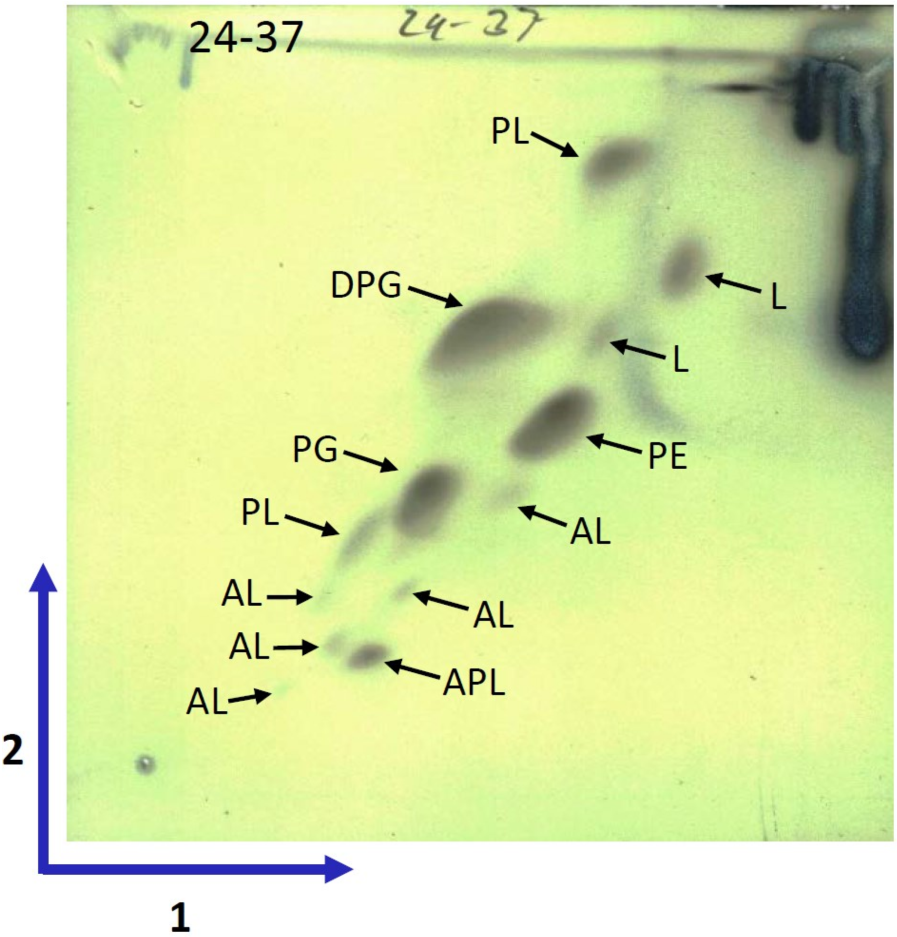

Figure S1. Total polar lipid profile of *Clostridium caseinilyticum* sp. nov. strain FAM 1755^T. DPG, diphosphatidylglycerol; PE, phosphatidylethanolamine; PG, phosphatidylglycerol; APL, aminophospholipid; AL, aminolipid; PL, phospholipid; L, lipid.
